# An *in Vitro* Comparison of Coronal Discolouration Caused by White Mineral Trioxide Aggregate, Theracal, Calcium-Enriched Mixture and Biodentine

**DOI:** 10.14744/eej.2020.83584

**Published:** 2022-01-07

**Authors:** Zohreh KHALILAK, Ehsan ESNAASHARI, Keivan SAATI, Delaram BINESHMARVASTI, Hazhir YOUSEFSHAHI, Mahsima NOBAKHT

**Affiliations:** From the Department of Endodontics (Z.K., D.B. 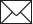 dlrmbm@yahoo.com, H.Y., M.N.), Islamic Azad University of Medical Science, Dental School, Tehran, Iran; Department of Endodontics (E.E.), Islamic Azad University Faculty of Dentistry, Tehran, Iran; Department of Operative Dentistry (K.S.), Islamic Azad University, Dental Branch, Tehran, Iran

**Keywords:** Biodentine, calcium-enriched mixture cement, mineral trioxide aggregate, tooth discoloration, TheraCal

## Abstract

**Objective:**

The use of calcium silicate-based cements has greatly increased in the past decade. This study compared coronal discolouration caused by white mineral trioxide aggregate (MTA), TheraCal (TC), calcium enriched mixture (CEM), and Biodentine (BD) on bovine enamel-dentin block.

**Methods:**

In this in vitro, experimental study, enamel-dentin blocks (7x7x3.5 mm) were cut out of 60 extracted sound bovine central incisors. A cavity (2.5 mm diameter and 1 mm depth) was created at the center of each block with 1 mm distance from the labial surface. The blocks were randomly divided into four experimental groups (n=12) of MTA, CEM, BD, and TC and two groups of positive (blood) and negative (empty) controls. After filling cavities with experimental materials, the surfaces of the materials were covered with composite resin. The colour parameters were measured using a spectrophotometer, before (T0) and 1 week (T1), 1 month (T2), and 6 months (T3) after the application of the materials. The data was analysed using repeated measures ANOVA and the Kruskal-Wallis test.

**Results:**

At 1 week and 1 month, a significant difference in ∆E was noted in the control groups compared to the experimental groups (P<0.001). The difference among the four cements was not significant (P=0.06) at 1 week but it was significant at 1 month. TC showed greater ∆E whereas BD showed lower ∆E in the six-month period (P<0.001). Colour change was significant over time in the MTA and TC groups (P<0.05).

**Conclusion:**

TheraCal caused greater discolouration in the bovine tooth blocks after 6 months, which may make it inappropriate for application in the aesthetic zone.

**HIGHLIGHTS**

• Different biocompatible materials could cause coronal discoloration.
• In short time different biocompatible materials are simillar in the means of coronal discoloration properties.
• in long term, Biocompatible materials could cause different degree of coronal discoloration.

## INTRODUCTION

Coronal discolouration is a common complication of the application of calcium silicate-based cements in vital pulp therapy or endodontically treated teeth, which is concerning for dental clinicians. An ideal endodontic cement should provide favourable aesthetic results in addition to optimal biological and mechanical properties (1, 2).

A wide range of dental materials including some intracanal medicaments such as Ledermix and Portland cement-based materials such as gray and white mineral trioxide aggregate (MTA) can cause tooth discolouration (1-5). Calcium-enriched mixture (CEM) is composed of calcium oxide, calcium phosphate, calcium carbonate, calcium silicate, calcium sulfate, calcium hydroxide, and calcium chloride (6, 7). It has clinical applications similar to those of MTA. CEM cement also polymerizes in aqueous environments but has easier clinical application, shorter setting time, and easier handling than MTA (ProRoot, Dentsply, Tulsa, OK, USA) (6, 7). Biodentine (Septodont, Saint Maur des Fosses, France) (BD) is another biocompatible calcium silicate-based cement with physical properties resembling those of dentine (8, 9). It has been proposed as a substitute to coronal and radicular dentine (8, 9). TheraCal (Bisco Inc., Schaumburg, IL, USA) (TC) is another biocompatible material. It has been designed for direct and indirect pulp capping treatments (10).

Several methods are available for the assessment of tooth discolouration (11). Thus, several electronic devices have been introduced for this purpose such as colourimeter, spectrophotometers, and digital cameras. However, digital cameras do not have the required accuracy and reproducibility for this purpose (12-14). Spectrophotometers have long been used for tooth colour assessment with high accuracy, reliability, and reproducibility (13-16). The overall colour change (∆E) can be calculated using the L*a*b* parameters. The calculation of ∆E according to the CIE L*a*b* colour space is the most common, comprehensive, and accurate method for the assessment of colour change in research studies and has ADA approval as well (17, 18). It should be noted that tooth discolouration can affect the patient’s quality of life (19, 20). Moreover, discolouration caused by the dental procedure can compromise patient satisfaction (21, 22).

Thus, this study sought to assess and compare coronal discoloration caused by MTA, CEM, TC, and BD 1 week, 1 month, and 6 months after their application. The null hypothesis was that there would be no significant difference in coronal discolouration caused by white MTA, CEM, TC, and BD at different time points.

## MATERIALS AND METHODS

This in vitro, experimental study evaluated 60 extracted sound bovine mandibular central incisors. Sample size was calculated to be 12 in each group according to a previous study [1], assuming a minimum ∆E of 8.3 and a standard deviation of 1.2 using Minitab software. The inclusion criteria were sound bovine mandibular central incisors with no cracks or carious lesions. Teeth were selected using convenience sampling. Debris and stains were removed using a scaler, and the teeth were then polished with a rubber cup and pumice paste. Tooth blocks composed of both enamel and dentin were cut out of each tooth using a diamond disc (Intensive; Grancia, Switzerland). Each block was measured 7 mm in length, 7 mm in width, and 3.5 mm in thickness. The dimensions of each block were measured using a caliper (Mitutoyo, Japan) to ensure the standardization of all the samples. Next, a cylindrical hole was created with 2.5 mm length and 1 mm depth at the center of each block using a diamond bur (Dentsply Maillefer) such that the hole had 1 mm distance from the labial surface of the tooth. To eliminate the smear layer, the blocks were immersed in 17% EDTA followed by 5.25% sodium hypochlorite, each for 1 min, and were then rinsed with saline. Afterward, the tooth blocks were randomly divided into four experimental groups of MTA (ProRoot, Dentsply,Tulsa, OK, USA) (n=12), CEM (Bionique Dent, Theran, Tehran, Iran) (n=12), BD (Septodont, Saint Maur des Fosses, France) (n=12), and TC (Bisco Inc., Schaumburg, IL, USA) (n=12) and two groups of positive (blood) (n=12) and negative (empty) (n=12) controls. The cements were mixed according to the manufacturers’ instructions and applied into the cavities using a Dycal applicator by an operator who was unaware of group distribution. After acquiring approval from the ethics committee and obtaining written informed consent, 10cc blood sample was obtained from a 24-year-old healthy male donor using an 18-gauge needle. The donor did not have any systemic disease.

Next, the cavities were temporarily sealed with a moist cotton pellet and the Coltosol temporary restorative material (Coltene, Germany). After the final setting of the cements (24 h later), all the cavities were restored with A1-shade light-cure flowable composite resin (Prim-Dent, USA). The curing process was performed according to the manufacturer’s instructions. Each block was separately immersed in a test tube containing artificial saliva and incubated at 37°C until evaluation time to simulate the oral environment.

### Colour assessment:

For colour assessment, the blocks were mounted in putty in prefabricated molds and their color parameters were measured at baseline (T0) and 1 week (T1), 1 month (T2), and 6 months (T3) after the application of the cements using a spectrophotometer (Shade Pilot; DeguDent International, Dentsply, Germany). Prior to the colour assessment of each sample, the spectrophotometer was calibrated using white and green tiles. For further accuracy, colour measurements for each sample were repeated three times and the mean of the three values was used for statistical analysis. The L*, a*, and b* colour parameters were measured as such and ∆E was calculated using the formula below:

### Statistical analysis

Repeated measures ANOVA was used to compare the ∆E of the six groups at different time points considering the material type as a between-subject factor (23). General comparisons were carried out using the Kruskal-Wallis test. All statistical analyses were performed using SPSS version 22 (SPSS Inc., IL, USA) at 0.05 level of significance.

∆E=((∆L^2^)+(∆α^2^)+(∆b^2^))^1/2^

## RESULTS

Table 1 shows the ∆E of the study groups at different time points. At T1, the positive control group had greater ∆E (20.8) whereas the negative control group showed lower ∆E (4.2), indicating the correct set-up of the study. The Kruskal-Wallis test revealed a significant difference in ∆E between the six groups at T1 compared to T0 (P<0.001). At T1, greater ∆E was noted in the TC group (13.2) whereas lower ∆E was noted in the BD group (11.6). However, ∆E was not significantly different in the four cement groups at T1 (P=0.06).

**TABLE 1.** ∆E of study groups at different time points

Group	1 week (T1)	1 month (T2)	6 months (T3)	P value
Negative control	4.2±2.1	7.3±3.2	8.6±3.7	<0.01
Positive control	20.8±7	33.8±18.6	34.8±5.6	<0.01
CEM cement	13.2±5.7	15.5±6.6	15.8±5.04	0.6
ProRoot MTA	12.73±6.4	15.1±8.6	18±2.8	<0.01
Biodentine	11.6±4.2	12±4.2	14.7±2.6	0.6
TheraCal	11.9±3.5	26.3±8.4	26.9±2.1	<0.001
P value	<0.001	<0.001	<0.001	

At T2, greater and lower ∆E values were noted in the positive (32.8) and negative (7.3) control groups, respectively. The difference in ∆E was significant in the six groups (P<0.001). Comparison of the four cement groups at this time point revealed greater ∆E in the TC group (26.3) and lower ∆E in the BD group (12); the Kruskal-Wallis test showed that this difference was statistically significant in the TC and positive control groups compared to the other groups (P<0.001).

At T2, the pairwise comparisons of the groups revealed that the difference in ∆E was significant between the MTA and BD groups (P<0.01) but not between the MTA and CEM groups (P=0.6). The difference in ∆E was also significant between the TC group and the other three cement groups (P<0.001).

Moreover, ∆E changed significantly at T3 (P<0.05) in the negative and positive control groups and also in the MTA and TC groups compared to T2 (P<0.05).

In the CEM cement and BD groups, ∆E did not significantly change at T3 compared to T2 (P=0.6).

## DISCUSSION

This study compared the coronal discolouration of bovine teeth caused by MTA, TC, CEM, and BD after 1 week, 1 month, and 6 months to find more suitable composition in terms of aesthetics. The results showed that coronal discolouration caused by BD was less intensive than that caused by the other three cements. However, discolouration caused by TC was greater among all the cements. All the materials caused discolouration after 6 months, which appears to be the time period required for the infiltration of coloured agents into the dentinal tubules.

Bismuth oxide and iron present in the composition of MTA were previously held responsible for tooth discoloration; thus, white ProRoot MTA without iron and bismuth oxide was developed. However, most studies failed to find a significant difference between gray and white MTA regarding the potential to cause tooth discoloration (1-5). Although unlike MTA, CEM, TC, and BD do not contain iron and bismuth oxide (6-10), the current results showed they also caused tooth discolouration. In contrast to our study, none of the samples used in Xin Li et al.’s study (24) which was treated with TC exhibited discolouration. Moreover, unlike our results, ProRoot MTA induced greater discolouration than TC after 7 and 70 days. This difference could be related to the method of color evaluation. Tooth discoloration was evaluated visually in Xin Li et al.’s study (24), while it was evaluated by spectrophotometry in the current study. It appears that the presence of other compounds such as barium strontium, and zirconium oxide (25) may be the cause of discolouration in TC. However, the exact cause and mechanism of discolouration by these cements have yet to be fully understood and require further investigations.

In this study, bovine central incisors were used due to their easy availability and the fact that they allow the preparation of larger blocks for the better simulation of clinical settings. Further, blocks with the same shape, size, and thickness were prepared of the teeth for the purpose of standardisation and to eliminate the confounding effect of tooth anatomy and morphology on the results. Moreover, cavities with the same size were created in blocks to apply similar amounts of cements in them (2).

Thomson et al. (16) used extracted human teeth and assessed discoloration caused by sealers with a digital camera. However, a spectrophotometer was used in the present study to assess discolouration since it is the most reliable tool for this purpose (13).

Akbari et al. (26) compared ∆E caused by gray and white MTA in the presence and absence of a bonding agent and assessed it using a colourimeter. They did not have a positive control group in their study and assessed color change after 6 months. However, we used a spectrophotometer, which is more accurate than a colourimeter, and assessed colour change 1 week, 1 month, and 6 months later.

Shahrami et al. in their study (27) stated that colour assessments should be performed in close time intervals to better reveal changes, which is similar to our findings. Lenherr et al. [1] evaluated the ∆E of bovine teeth after using a number of endodontic sealers and endodontic cements such as white and gray MTA and reported that white MTA caused a ∆E smaller than blood, which is similar to our findings at 6 months. Bortoluzzi et al. (28) performed a clinical study and used gray and white MTA in traumatised discoloured central incisors. They reported that gray MTA did not cause tooth discolouration. However, they did not assess color change in the long-term. It should be noted that discolouration takes a longer time to occur if the smear layer is not removed. Felman and Parashos (29) compared coronal discolouration caused by gray and white MTA in the short-term (35 days) and showed that the both cements caused discolouration after the period. They used premolar teeth and filled the entire root canal and the pulp chamber with the cement, which is different from our methodology and can exaggerate results. Moreover, they assessed colour change using digital photography and Photoshop software, which are not highly accurate compared to spectrophotometry used in our study.

Yoldas et al. (30) evaluated 40 bovine incisors to assess the discolouration potential of endodontic cements such as BD after 1 day, 1 week, 1 month, and 1 year. They reported increased ∆E in all the samples at 1 year. BD caused less discolouration than blood, which is in agreement with our findings. Marconyak et al. (31) evaluated mandibular third molars to assess discolouration caused by BD, ProRoot MTA, and some other endodontic cements. They measured color change after 1, 7, 30, and 60 days and reported that BD caused less discoloration than ProRoot MTA. This result is in accord with our findings at 1 month.

Esmaeili et al. (32) assessed 40 maxillary central incisors to compare discoloration caused by CEM and white MTA. They stated that CEM was a suitable cement for use in the aesthetic zone due to its insignificant discolouration potential. Arman et al. (23) evaluated 32 extracted human teeth to assess discoloration caused by ProRoot MTA and CEM after 1 week, 1 month, and 6 months. They reported no significant difference in discolouration caused by the two materials at 6 months, which is in agreement with our results. Parsons et al. (33) reported minimum and maximum colour change at 0-3 months and 6 months, respectively, which is in line with our results. Vallés et al. (34) evaluated the colour change of BD *in vitro* and reported that BD had optimal colour stability at 6 months. In our study, BD caused the lowest colour change among the tested cements. Ramos et al. (35) evaluated 28 premolar teeth to assess discolouration caused by BD and white MTA after 1 year. They reported no significant difference in ∆E between the two cements at 1 year. In our study, the difference in ∆E between these two cements was significant at 6 months. The difference between their results and ours may be due to different assessment time points. The difference between the two cements could have been insignificant if we had assessed ∆E at 1 year. Eghbal et al. (36) evaluated the ∆E of enamel of 12 teeth after the application of CEM and white MTA. They assessed colour change using a digital camera. They reported that after 28 days, CEM caused less discolouration than white MTA. Although they used a digital camera, their results are in agreement with ours.

It should be noted that this study had an in vitro design and was conducted on bovine teeth. The oral environment cannot be perfectly simulated in vitro. Moreover, bovine teeth have structural differences with human teeth, which limit the generalisation of results to human teeth. Future in vitro studies on human teeth followed by clinical studies are required to obtain more reliable results in this respect.

## CONCLUSION

Six months after treatment, the TC group demonstrated the greatest discoloration and the Biodentine group demonstrated the least discoloration. According to the results, it is highly recommended to further investigate how the current cements affect human teeth to find more evidence on side effects of the cements on tooth color.
